# The N. Y. Code of Ethics

**Published:** 1883-02-20

**Authors:** 


### *THE NEW YORK CODE OF ETHICS.*

Certain of the county medical societies in New York State have taken action against the new code, while others sanction.

The Society of the County of Kings (Brooklyn) having once instructed their delegates to the State Society to vote against the new code, have rescinded that action and instructed their delegates to vote for the new code.

